# Genetic Ablation of CD68 Results in Mice with Increased Bone and Dysfunctional Osteoclasts

**DOI:** 10.1371/journal.pone.0025838

**Published:** 2011-10-03

**Authors:** Jason W. Ashley, Zhenqi Shi, Haibo Zhao, Xingsheng Li, Robert A. Kesterson, Xu Feng

**Affiliations:** 1 Department of Pathology, University of Alabama at Birmingham, Birmingham, Alabama, United States of America; 2 Department of Internal Medicine, University of Arkansas for Medical Sciences, Little Rock, Arkansas, United States of America; 3 Department of Nutrition Sciences, University of Alabama at Birmingham, Birmingham, Alabama, United States of America; 4 Department of Genetics, University of Alabama at Birmingham, Birmingham, Alabama, United States of America; Medical College of Georgia, United States of America

## Abstract

CD68 is a member of the lysosome associated membrane protein (LAMP) family that is restricted in its expression to cells of the monocyte/macrophage lineage. This lineage restriction includes osteoclasts, and, while previous studies of CD68 in macrophages and dendritic cells have proposed roles in lipid metabolism, phagocytosis, and antigen presentation, the expression and function of CD68 in osteoclasts have not been explored. In this study, we investigated the expression and localization of CD68 in macrophages and osteoclasts in response to the monocyte/macrophage-colony stimulating factor (M-CSF) and the receptor activator of NF-κB ligand (RANKL). We found that M-CSF stimulates CD68 expression and RANKL alters the apparent molecular weight of CD68 as measured by Western immunoblotting. In addition, we explored the significance of CD68 expression in osteoclasts by generating mice that lack expression of CD68. These mice have increased trabecular bone, and *in vitro* assessment of CD68^−/−^ osteoclasts revealed that, in the absence of CD68, osteoclasts demonstrate an accumulation of intracellular vesicle-like structures, and do not efficiently resorb bone. These findings demonstrate a role for CD68 in the function of osteoclasts, and future studies will determine the mechanistic nature of the defects seen in CD68^−/−^ osteoclasts.

## Introduction

CD68, also known as macrosialin, is a heavily glycosylated LAMP family member that is commonly used as a histological marker of macrophage lineage cells. Indeed, CD68 expression can be found in the resident macrophages of multiple tissues such as microglia in the brain, Kupffer cells in the liver, and bone marrow macrophages (BMMs) [1]–[3]. Furthermore, immunohistochemical staining of bone tissues has demonstrated expression of CD68 in osteoclasts, which are multinucleated, bone-resorbing giant cells of monocyte/macrophage origin [3], [4]. Infiltration of CD68-positive cells is also used clinically as a marker of inflammation and tumor progression [5]–[8]. The apparent monocyte/macrophage lineage specificity of CD68 expression has not only led to its routine use in histological identification of macrophages, but to the proposed use of the CD68 promoter to specifically direct transgene expression in both *in vitro* and *in vivo* model systems and gene therapy [9]–[11]. Though both CD68 protein and gene have been and continue to be used as tools in both research and clinical settings, studies of the physiological function(s) of CD68 remain inconclusive.

Despite the lack of a specific, defined role for CD68 in cells, studies of the characteristics and regulation of the CD68 gene and protein have inspired many of the early studies into its function. Ramprasad et al. demonstrated via ligand blotting and affinity purification experiments that CD68 is capable of binding oxidized low density lipoprotein (oxLDL) and can be detected on the surface of macrophages [12]–[14]. This oxLDL binding property was found by multiple groups, and, recently, the oxLDL binding properties of CD68 were exploited in a pre-clinical model wherein atherosclerotic lesion progression was retarded by application of a soluble Fc receptor-CD68 fusion protein [15], [16]. The binding of oxLDL by CD68 appears to be independent of associated sugar moieties as even deglycosylated CD68 retains much of its oxLDL binding affinity. CD68 not only binds oxLDL, but its expression also appears to be upregulated by oxLDL [17]. de Beer et al. found that levels of CD68 in the livers of mice fed a high fat diet were increased, and a direct effect of lipid on macrophage expression of CD68 was confirmed by Llaverias et al. [18], [19]. These studies of CD68’s oxLDL binding affinity and its expression on the cell surface suggested a role for CD68 in the uptake of oxLDL, and, indeed, antibody blockage of CD68 on PMA-stimulated THP-1 monocytes resulted in reduced binding and uptake of oxLDL [13]. However, dsRNA-mediated silencing of CD68 expression in both primary peritoneal macrophages and macrophage-like RAW264.7 cells failed to reduce oxLDL binding by either cell type [18]. In addition, it was recently determined via examination of peritoneal macrophages from mice lacking expression of CD68 that neither oxLDL uptake nor microbe phagocytosis is dependant on CD68 expression [20]. Thus, despite its accepted oxLDL binding properties, the specific role of CD68 remains controversial, and there has been no further demonstration of the physiological significance of CD68 expression in any cell type.

Given that most studies of CD68 have focused on macrophages and macrophage-like cell lines, we set out to examine CD68’s expression by osteoclasts, its regulation by the osteoclastogenic cytokines M-CSF and RANKL, and the consequences of its genetic ablation. Here we report that loss of CD68 results in morphological and functional defects in osteoclasts *in vitro* that result in increased bone *in vivo*.

## Results

### CD68 expression during osteoclastogenesis

**Figure 1:**
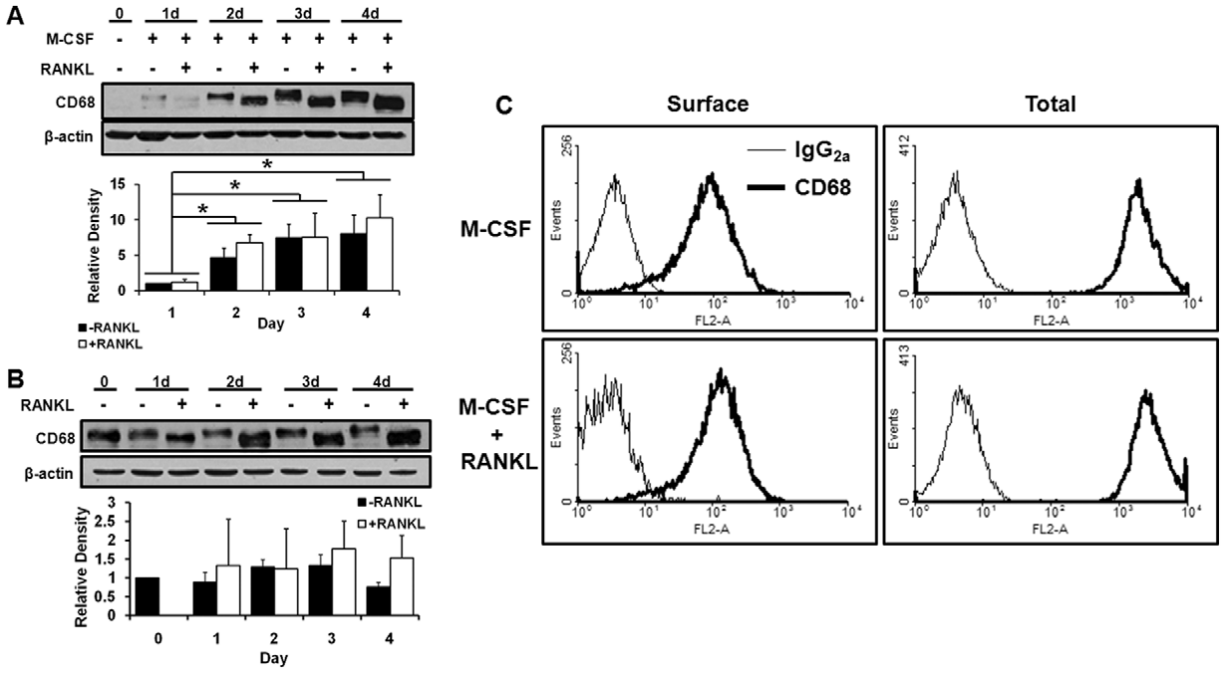
CD68 is expressed by macrophages and osteoclasts. (A) CD68 and β-actin expression in mouse bone marrow suspension cells treated with 44 ng/mL M-CSF +/− 100 ng/mL RANKL for indicated days (d). M-CSF increases expression of CD68 in a time dependant manner, and RANKL ligand induces an accelerated gel migration rate. Image is representative of 3 independent experiments. Quantification of relative band density is aggregate of 3 independent experiments; data shown is mean + standard deviation. (B) CD68 and β-actin expression in RAW264.7 cells with or without 100 ng/mL RANKL treatment for indicated times. RAW264.7 cells have constitutive M-CSF-stimulated signaling and continuous expression of CD68. RANKL induces similar changes in gel migration as those seen in primary macrophages. Image is representative of 3 independent experiments. Quantification of relative band density is aggregate of 3 independent experiments; data shown is mean + standard deviation. (C) Flow cytometry histograms of BMMs with 3-day treatments of 44 ng/mL M-CSF +/– 100 ng/mL RANKL with (Total) and without (Surface) permeablization with .5% saponin. CD68 can be detected on the surface of BMMs, and neither surface nor total detectible levels of CD68 are altered by addition of RANKL. Image is representative of 2 independent experiments.

We first assessed the expression of CD68 by BMMs and osteoclasts. To this end, we collected whole cell lysates from primary mouse BMMs cultured for varying periods of time in M-CSF alone, which maintains the macrophage characteristics of the cells, and BMMs cultured with M-CSF and RANKL, which induces differentiation of BMMs to osteoclasts. We found that freshly isolated non-adherent bone marrow mononuclear cells do not express detectible levels of CD68, and addition of M-CSF stimulated expression of CD68 in a time-dependant manner (Fig. 1A). Interestingly, while addition of RANKL did not result in significantly altered levels of CD68 compared to M-CSF alone, RANKL treatment reduced CD68’s apparent molecular weight as measured by its migration rate during polyacrilamide gel electrophoresis followed by Western blotting. Similar to primary BMMs, RAW264.7 cells, which are self-sufficient in their M-CSF receptor signaling, constitutively express CD68, and addition of RANKL resulted in a comparable shift in CD68’s migration rate with no significant change in expression (Fig. 1B). CD68 can be found on the cell surface of macrophages, and this RANKL-induced form of CD68 may be subject to altered surface localization [21], [22]. To determine whether the RANKL-induced form of CD68 can still be detected on the surface of BMMs, we analyzed primary BMMs treated for 72 hours with either M-CSF alone or M-CSF and RANKL via flow cytometry. We found that BMMs cultured with M-CSF alone express detectible levels of CD68 on their surface, and RANKL treatment does not appear to alter this surface expression (Fig. 1C–“Surface”). CD68 expression was also detected intracellulalry by permeablizing cells prior to staining (Fig. 1C–“Total”).

### Cellular localization of CD68 in osteoclasts

**Figure 2:**
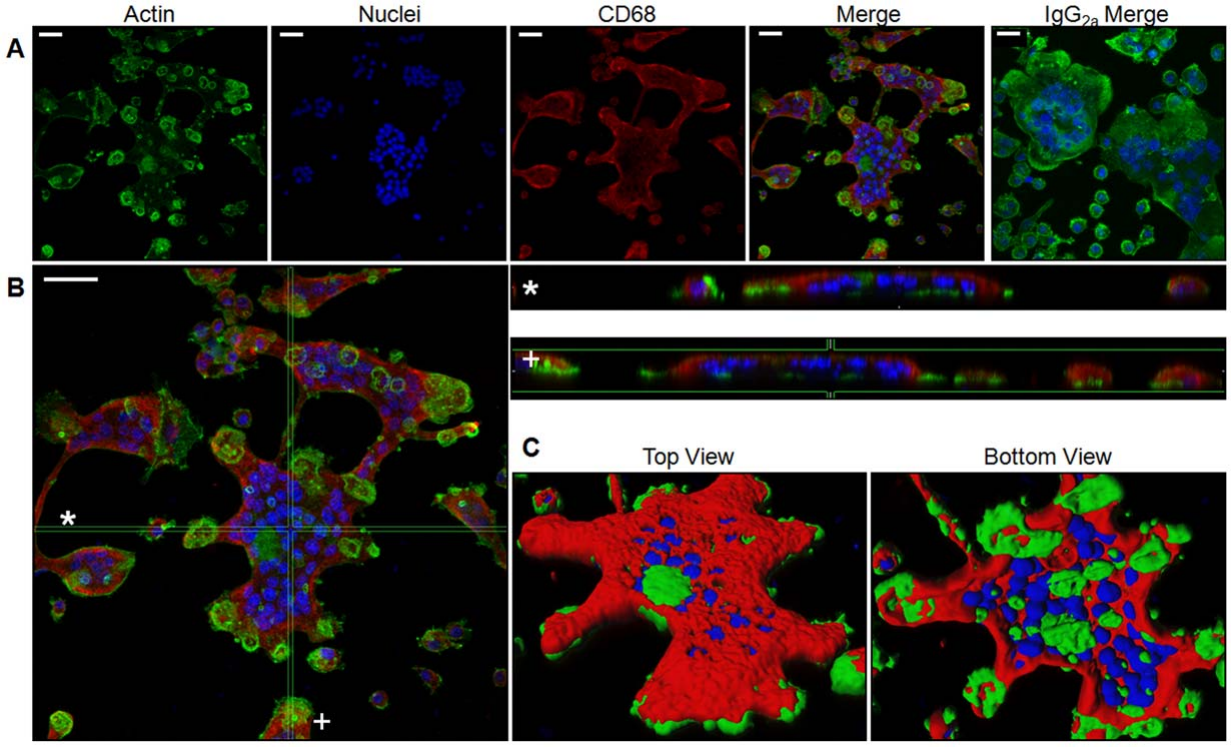
CD68 can be found in a dome-like pattern in osteoclasts cultured on bovine cortical bone slices. (A) BMMs were seeded onto bone slices with 44 ng/mL M-CSF and 100 ng/mL RANKL and differentiated into osteoclasts over 4 days. Cells were fixed with 4% paraformaldehyde/PBS and stained with Alexa-488-conjugated phalloidin (actin, green), Hoescht (nuclei, blue), and either anti-CD68 (CD68, red) or rat non-immune IgG_2a_ (IgG_2a_ Merge) antibody followed by Alexa-647-conjugated anti-Rat IgG. Scale bars for CD68 staining are 40μm; scale bar for IgG_2a_ staining is 20μm. Images are representative of 3 independent experiments. (B) Enlarged merge image from A. * and + indicate corresponding XZ and YZ cross sectional images, respectively. Scale bar is 40μm. Image is representative of 3 independent experiments. (C) 3-D reconstruction of osteoclast cross sectioned in B with actin in green, nuclei in blue, and CD68 in red.

Our own immunoblotting and published tissue immunohistochemical studies have revealed expression of CD68 by osteoclasts. We next sought to determine the intracellular distribution of CD68 in mature, bone-adherent osteoclasts by performing immunofluorescent staining of osteoclasts differentiated on bovine cortical bone slices. Following staining with Alexa-488-conjugated phalloidin for actin (green), Hoechst for nuclei (blue), and either anti-CD68 antibody or non-immune Rat IgG_2a_ (red), cells were visualized using confocal microscopy (Fig. 2). Staining revealed multiple nuclei and actin rings which are morphological features of mature osteoclasts and intense localization of CD68 around the periphery of the osteoclasts (Fig. 2A). CD68 could also be detected, though less intensely, towards the central regions of the cell. Visualization of osteoclasts along the Z-axis revealed a vertical concentration of CD68 at the osteoclast periphery with a more apical localization towards the center of the cell (Fig. 2B). Three-dimensional reconstruction of imaged osteoclasts confirmed this dome-like distribution with CD68 detected near both the bone-apposed, basolateral, and apical surfaces along the cell periphery, but only near the apical surface elsewhere (Fig. 2C).

### Generation of CD68^−/−^ mice lacking expression of CD68

**Figure 3:**
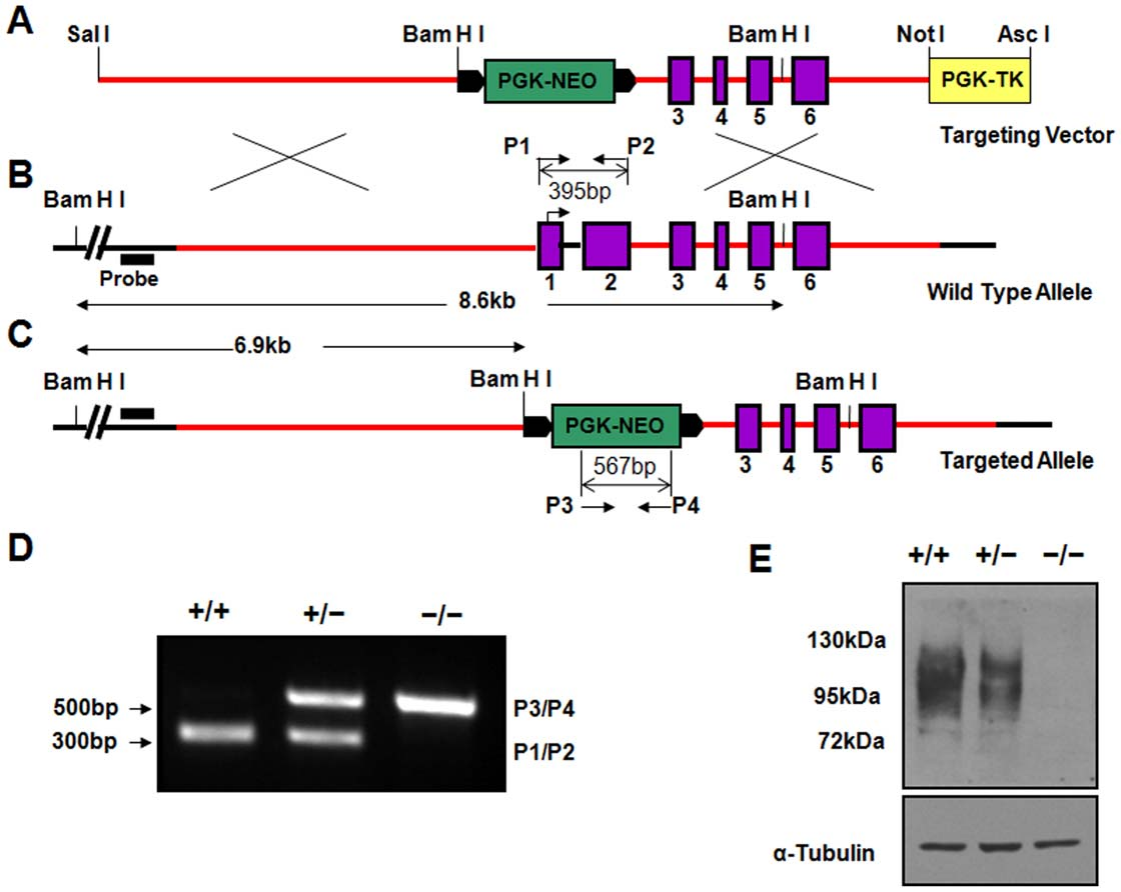
BMMs from CD68^−/−^ mice lack expression of CD68. (A) Vector diagram with a neomycin phosphotransferase expression cassette (PGK-NEO) flanked by sequences with homology to targeted genomic sequence. A thymidine kinase expression cassette (PGK-TK) lies outside the homology region of vector. (B) Structure of targeted wild type allele. A southern blot probe can hybridize to a genomic sequence outside of the homology region. P1 and P2 are primers that specifically amplify the sequence of CD68 targetted for replacement. (C) Recombined allele with exons 1 and 2 of CD68 gene replaced with PGK-NEO. Properly targeted recombined alleles do not contain the thymidine kinase expression cassette. P3 and P4 are primers that specifically amplify a region of PGK-NEO. (D) Tail tip extracts from each of three resultant genotypes were subjected to genotyping PCR using P1, P2, P3, and P4. Each genotype produced a unique pattern of PCR products. (E) Lysates from BMMs from each genotype cultured with 220 ng/mL M-CSF were immunoblotted with antibodies against CD68 and α-Tubulin. While expression of CD68 was seen in +/+ and +/− BMMs, no CD68 could be detected in lysates from −/− BMMs.

Detection of CD68 on the surface of BMMs and its distribution in osteoclasts prompted us to initiate deeper functional studies of this protein. To further explore the role of CD68 in bone specifically and elsewhere in general, we generated a line of mice that lack expression of CD68. Fig. 3A–C detail the strategy of homologous recombination within C57Bl/6J mouse embryonic stem (ES) cells to generate the CD68 knockout allele. This recombination event replaces exon 1, which contains CD68 start codon, and part of exon 2 with a neomycin phosphotransferase expression cassette (Fig. 3A–B). Male chimeras generated by injecting the correctly targeted ES cells into albino C57Bl/6J blastocysts were mated with albino C57Bl/6J females, and black-coated offspring were genotyped using primers specific to the wild type (Fig. 3B) and knockout alleles (Fig. 3C) to identify CD68^+/−^ heterozygotes. Male and female heterozygotes were then mated to produce CD68^+/+^, CD68^+/−^, and CD68^−/−^ offspring as determined by PCR (Fig. 3D). CD68^−/−^ mice are viable and are born near to expected Mendelian frequencies (Table 1). Similar to mice generated by Li et al., we observed no obvious behavioral or gross phenotypic abnormalities in the CD68^−/−^ animals [20]. We confirmed loss of CD68 expression in CD68^−/−^ mice by performing immunoblots on BMM protein extracts from mice of each genotype (Fig. 3E).

**Table 1:** Mouse Birth Ratios.

Sex	+/+	+/−	−/−	Sex/Total
Male	0.22	0.55	0.24	0.52
Female	0.18	0.5	0.32	0.48

### Bone phenotype of CD68^+/+^, ^+/−^, and ^−/−^ mice

**Figure 4:**
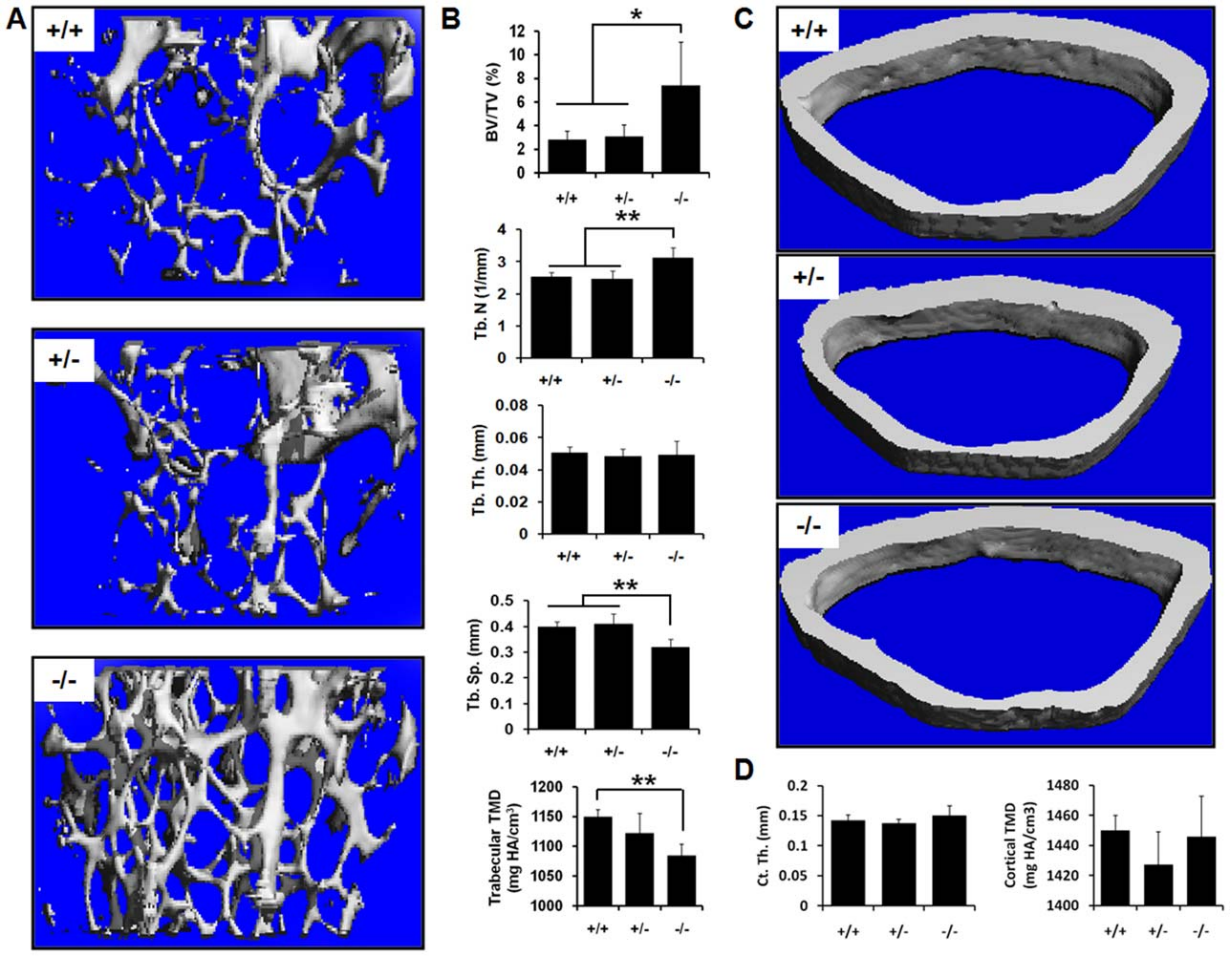
CD68^−/−^ mice have increased trabecular bone and decreased trabecular tissue mineral density. (A) Representative trabecular µCT images from 6-month-old female mice of each genotype (5 mice per group). (B) µCT analysis of distal femoral trabecular bone revealed that CD68^−/−^ mice have increased bone volume (BV/TV), increased trabecular number (Tb. N), decreased trabecular spacing (Tb. Sp.) and decreased trabecular tissue mineral density (Trabecular TMD). There was no significant difference in trabecular thickness (Tb. Th.). (C) Representative cortical µCT images from each genotype. (D) Quantification of measured parameters. There was no significant difference between genotypes in either cortical thickness (Ct. Th.) or cortical tissue mineral density (Cortical TMD). *, p<.05; **, p<.01. Data presented are means + standard deviation.

**Figure 5:**
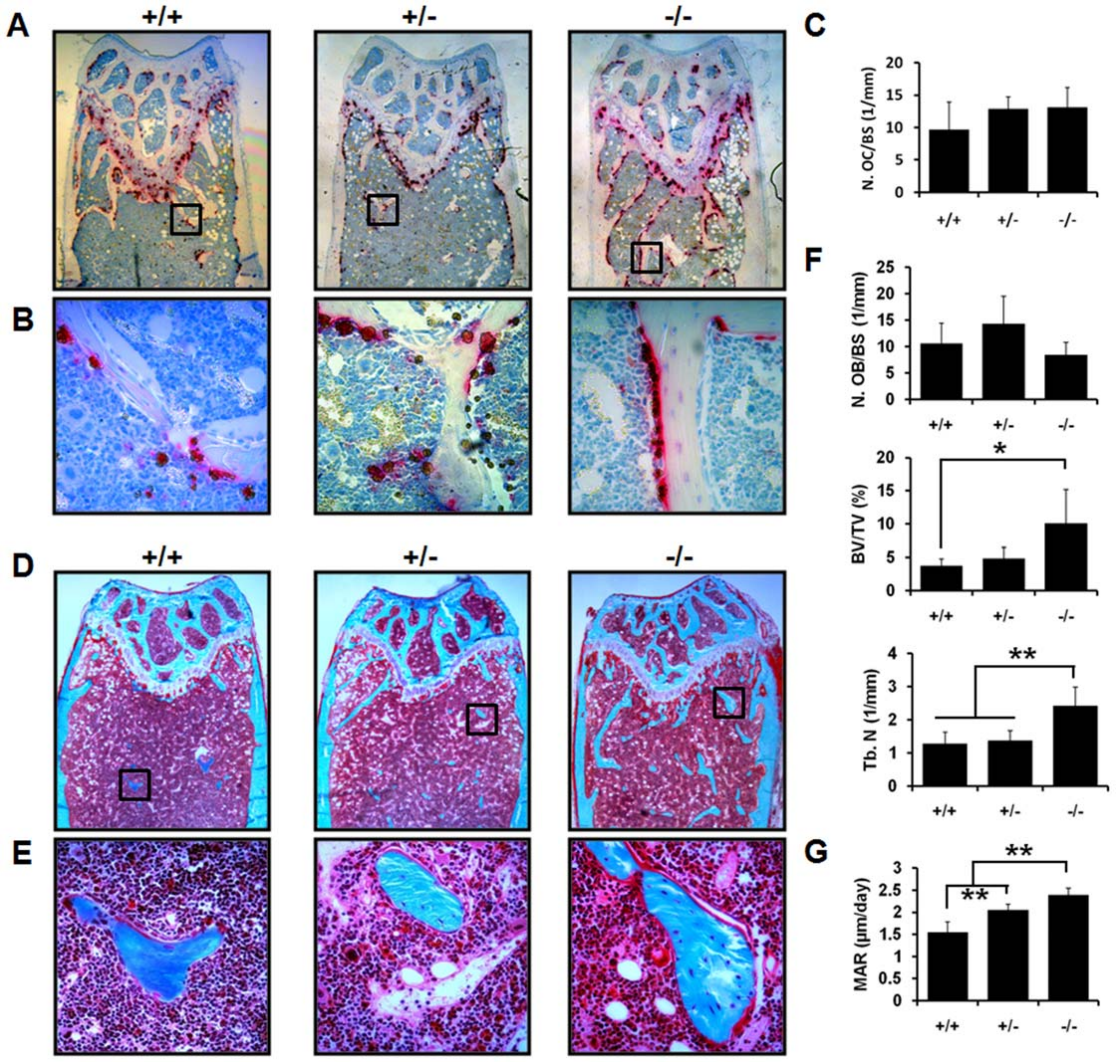
Histological and histomorphometric analysis of CD68^+/+^, ^+/−^, and ^−/−^ mice. (A) Representative formalin-fixed, paraffin-embedded histological sections from each genotyped stained for TRAP activity and counterstained with hematoxylin at 40X magnification. (B) 400X magnification of area defined in (A). (C) Quantification of number of osteoclasts per bone surface (N. OC/BS). There was no significant difference in N. OC/BS between genotypes. (D) Representative 70% ethanol-fixed, plastic-embedded histological sections from each genotype stained with Goldner’s trichrome at 40X magnification. (E) 400X magnification of area defined in (D). (F) Histomorphometric analysis of trichrome-stained sections. There was no significant difference between genotypes in numbers of osteoblasts (N. OB/BS) per bone surface. There was a significant increase in bone volume per total volume (BV/TV) in CD68^−/−^ mice compared to CD68^+/+^ animals and trabecular number (N. Tb.) in CD68^−/−^ mice compared to CD68^+/+^ and CD68^+/−^ animals. (G) Quantification of mineral apposition rate (MAR) via analysis of calcein double labeling. The MAR was significantly higher in CD68^−/−^ mice compared to both CD68^+/+^ and CD68^+/−^ animals, and MAR was significantly higher in CD68^+/−^ mice compared to CD68^+/+^ animals. *, p<.05; **, p<.01. Data presented are means + standard deviation.

To determine the consequences of genetic ablation of CD68 on the bone microarchitecture and tissue mineral density (TMD), we assessed the distal femurs of 6-month-old female mice using micro-computed tomography (μCT) and histology. TMD is the measure of mineral content of bone tissues only, as compared to bone mineral density (BMD), which includes surrounding non-bone tissues. This makes TMD a more accurate measurement of the mineral density of specific microarchitectureal structures such as trabecular and cortical bone[23]. Quantitative µCT data revealed that, compared to CD68-expressing mice, mice lacking CD68 have increases in bone volume (BV/TV) and trabecular number (Tb. N) with a concurrent decrease in trabecular spacing (Tb. Sp.)(Fig. 4A–B). Interestingly, there was a significant reduction in trabecular TMD in CD68^−/−^ mice compared to CD68^+/+^ mice with CD68^+/−^ trending towards lower trabecular TMD (Fig. 4B); this is intriguing because, while there is more trabecular bone in CD68 deficient animals, the mineralization appears to be abnormal. This decrease in TMD was not seen in cortical bone, however, nor was cortical thickness altered (Ct. Th.) (Fig. 4C–D). Histological examination of decalcified, TRAP-stained femur sections similarly showed increased trabeculae in CD68^−/−^ mice and abundant bone-adherent osteoclasts in all three genotypes (Fig. 5A–B) with no significant difference in osteoclast numbers (N. OC/BS) (Fig. 5C). These findings of increased trabecular bone without a corresponding decrease in osteoclasts suggest that loss of CD68 negatively impacts osteoclast function without reducing osteoclast numbers *in vivo*.

**Figure 6:**
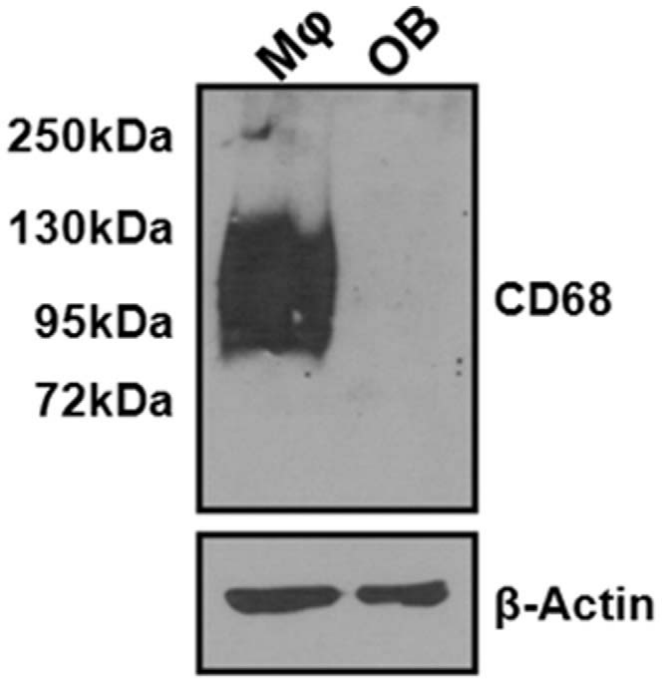
CD68 is not expressed by osteoblasts. CD68 and β-actin expression in mouse BMMs(Mϕ) cultured with 220 ng/mL M-CSF and cultured mouse calvarial osteoblasts (OB) was determined by Western immunoblotting. While expression of CD68 is high in BMMs, expression of CD68 could not be detected in osteoblasts.

As with the paraffin-embedded, TRAP-stained sections, plastic-embedded trichrome-stained sections demonstrated increased trabecular bone (Fig. 5D–E), and static histomorphometry values showed concordance with previous µCT-measured parameters (Fig. 5F-BV/TV and Tb. N). The decrease in trabecular TMD suggested a potential defect in osteoblast function, but quantification of osteoblast numbers showed no significant differences between the three genotypes (Fig. 5F–N. OB/BS). Furthermore, both CD68^+/−^ and CD68^−/−^ mice showed significant increases in mineral apposition rate (MAR) as measured by dynamic histomorphometry of double calcein-labeled sections (Fig. 5G). We found that wild type osteoblasts do not express CD68, which suggests that the observed changes in mineralization are likely due to indirect effects of CD68 ablation on osteoblast function (Fig. 6).

### *In vitro* assessment of CD68^+/+^, ^+/−^, and ^−/−^ osteoclast activity

**Figure 7:**
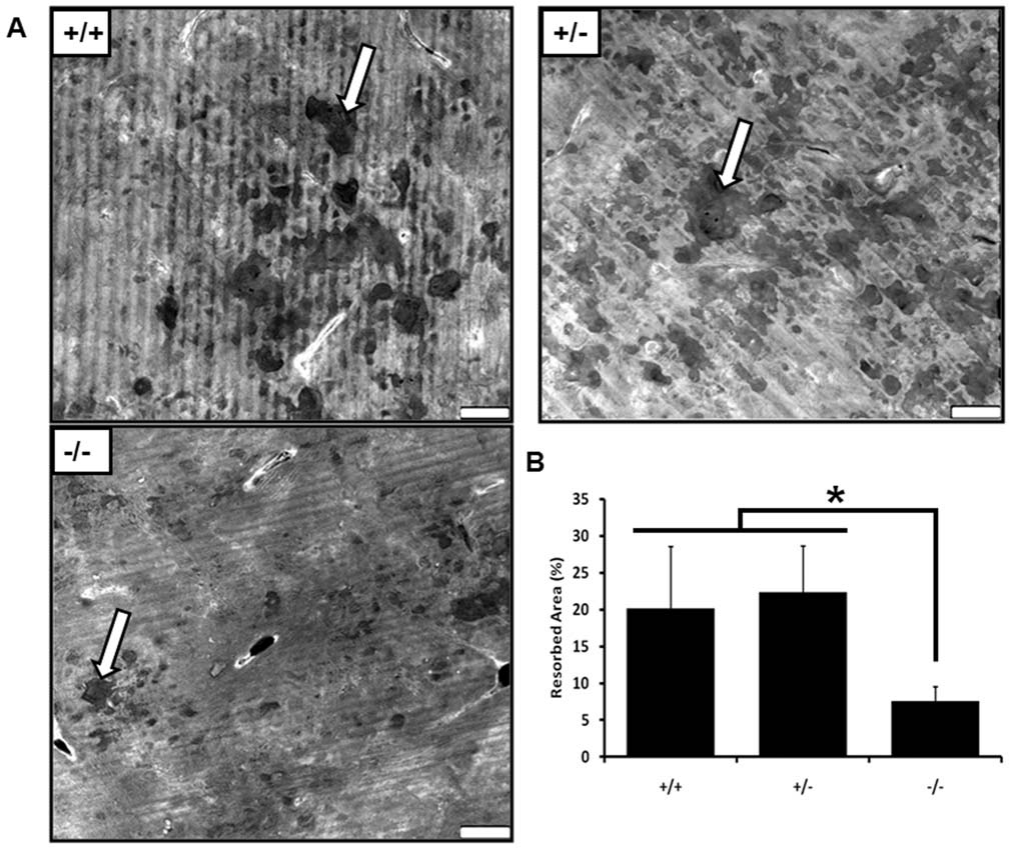
CD68^−/−^ osteoclasts do not efficiently resorb bone. BMMs from CD68^+/+^, ^+/−^, and ^−/−^ mice were seeded onto bovine cortical bone slices and differentiated into osteoclasts over 4 days. Differentiated osteoclasts were allowed to resorb the slices for an additional 3 days. (A) Representative images of resorbed bone slices generated using laser scanning confocal microscopy. A pit from each image is marked with an arrow. Scale bars are 70μm. (B) Quantification of resorbed area. Data represented as means + standard deviation. *, p<.001. 3 visual fields each from 3 separately resorbed bone slices were assessed. Data presented are means + standard deviation. Images and data are representative of 2 independent experiments.

Given the increase in trabecular bone seen *in vivo* following CD68 knockout, we next sought to determine the bone resorption efficiency of osteoclasts differentiated *in vitro*. BMMs were seeded onto bovine cortical bone slices with M-CSF and RANKL and allowed to differentiate into mature osteoclasts over 4 days. After an additional 3 days of resorption, cells were removed from the bone slices, and pits were imaged using confocal microscopy (Fig. 7A). We found that CD68^−/−^ osteoclasts resorbed a significantly smaller area than CD68-expressing osteoclasts by quantification of images generated from multiple bone slices (Fig. 7B). This confirmed our suspicion that CD68^−/−^ osteoclasts are less efficient at bone resorption than their CD68-expressing counterparts.

### *In vitro* morphological characterization of CD68^+/+^, ^+/−^, and ^−/−^ osteoclasts

**Figure 8:**
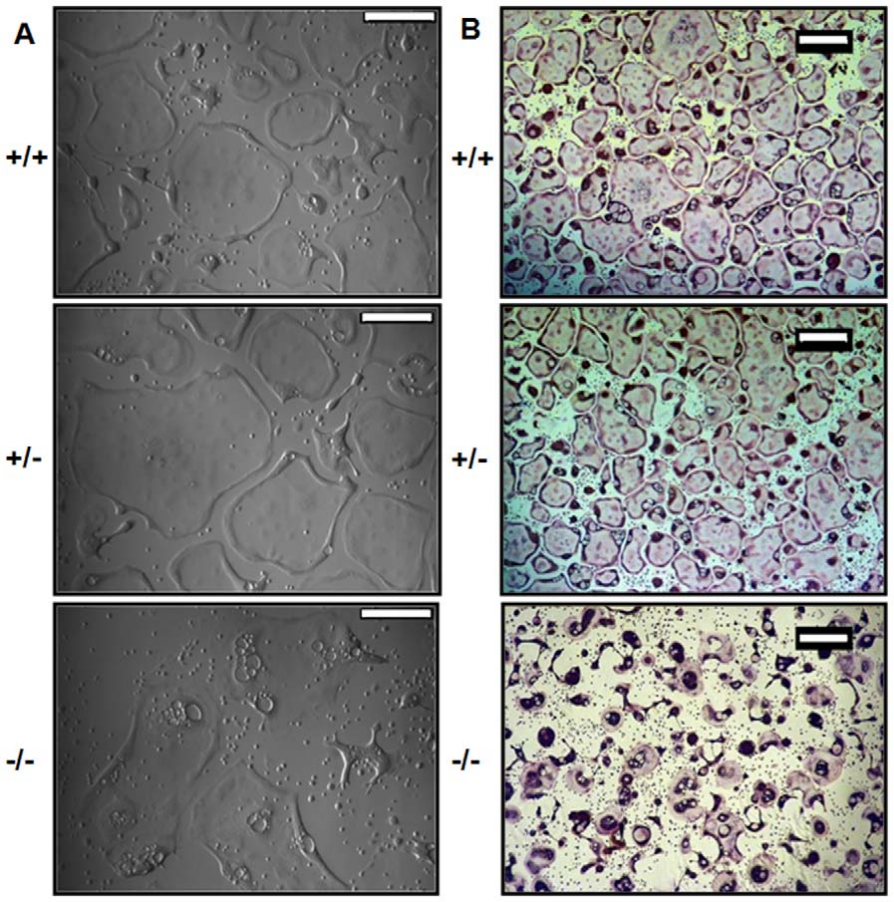
CD68^−/−^ have abnormal morphology. (A) Prior to TRAP staining, osteoclasts of all three genotypes were of relatively similar size. CD68^−/−^ osteoclasts demonstrated intracellular vacuole-like structures that were not present to such an extent in CD68-expressing cells. Scale bars are 100μm. Images are representative of 3 independent experiments. (B) During the fixation process (fixative: 25 mL citrate solution [18 mM citric acid, 9 mM sodium citrate, 12 mM sodium chloride, pH 3.6], 68 mL acetone, 8 mL 37% formaldehyde), many CD68^−/−^ osteoclasts were reduced in size and partially detached from the culture substrate resulting in a smaller size following TRAP staining. Scale bars are 200μm. Images are representative of 3 independent experiments.

In addition to preparing osteoclasts on bone slices to measure their resorption efficiency, we differentiated osteoclasts from precursors of each genotype in tissue culture plates to assess their *in vitro* morphology. Following differentiation, CD68^−/−^ osteoclasts appear as well spread as CD68-expressing osteoclasts, but osteoclasts lacking CD68 demonstrate an intracellular accumulation of vesicle-like structures that occur only rarely in CD68-expressing osteoclasts (Fig. 8A). Following TRAP staining, we found that many of the previously well-spread CD68^−/−^ osteoclasts had reduced much of their membranes to spindle-like projections resulting in abnormally shaped cells (Fig. 8B). Furthermore, we found that CD68^−/−^ osteoclasts were more susceptible to EDTA-induced lifting than CD68-expressing cells. As measured by soluble TRAP activity assay, while 53±4% of wild type osteoclasts remain attached to culture plates after a 10 minute treatment with 0.02% EDTA, only 8±4% of CD68^−/−^ osteoclasts remained attached after a similar treatment (p<.001). These morphological and lifting data indicate that CD68 is necessary for the health of osteoclasts, and, in its absence, osteoclasts acquire dysfunctions that are manifest in the form of aberrant morphology and reduced adhesion to culture substrates.

## Discussion

Although CD68 is routinely used as a histological marker of macrophage lineage cells, its specific function(s) in these cells remain undefined. Multiple studies have demonstrated CD68’s oxLDL binding affinity, but its expression appears to have little impact on the uptake of oxLDL [13], [16], [18]. There was evidence in favor of a role in oxLDL uptake including surface expression of CD68 as well as its rapid recycling between the intracellular/endosomal compartment and cell surface [14]. Furthermore, initial antibody-blockade studies on PMA-differentiated THP-1 macrophages showed that inhibition of CD68 reduced binding and uptake of oxLDL [13]. Nevertheless, RNAi studies in peritoneal macrophages and macrophage-like RAW264.7 cells, however, suggested that CD68 inhibition does not reduce oxLDL uptake, and forced expression of CD68 in COS-7 kidney cells did not increase the ability of these cells to take up oxLDL [18]. Further evidence against a role for CD68 in oxLDL uptake can be seen in CD68^−/−^ peritoneal macrophages which take up oxLDL as efficiently as CD68-expressing cells [20]. Thus, it appears that CD68 does not play an indispensible role in oxLDL uptake in macrophages. With the apparent resolution of this controversy, the question of CD68’s role in cells, nevertheless, remains unanswered. To date, there has been no demonstration of cellular dysfunction due to CD68 inhibition, nor has there been prior evaluation of the significance of CD68 expression by cells other than macrophages and myeloid dendritic cells.

In this study, we examined the expression and localization of CD68 in bone marrow macrophages and osteoclasts and demonstrated that CD68 expression is critical to the normal morphology and function of the osteoclasts. This represents the first example of cellular dysfunction due to CD68 inhibition. Consistent with its status as a marker of macrophage lineage cells, we found expression of CD68 in both BMMs and osteoclasts, but not osteoblasts. Though CD68 expression levels in BMMs and osteoclasts were comparable, the migration rate CD68 from cells treated with RANKL was accelerated, suggesting an alteration in its glycosylation. The nature and significance of this altered glycosylation has not been defined, though its consistent appearance in both BMMs and macrophage-like RAW264.7 cells is compelling. It has been shown that CD8’s glycosylation is subject to alteration in response to inflammatory stimuli [21]. Phagocytosis, in particular, induces a change from a predominantly core 1 pattern of o-glycosylation to a core 2 state in peritoneal macrophages [22]. This “phagocytic glycoform” of CD68 has also been detected in BMMs. This alteration does not appear to have an effect on surface expression, however, as comparable amounts of CD68 can be detected on the surface of macrophages treated with either M-CSF alone or M-CSF and RANKL. This altered glycoform may, however, be related to the function of CD68 in osteoclasts, and future studies should explore this. Further examining localization, we found that CD68 has a dome-like distribution in osteoclasts cultured on bone slices. This pattern arises from a concentration of CD68 along the Z-axis of the osteoclast periphery with a more exclusively apical distribution elsewhere.

In order to examine the significance of CD68 expression in osteoclasts specifically and the consequences of its ablation in whole animals in general, we used targeted genomic recombination to generate mice that lack expression of CD68. We found that CD68^−/−^ pups appear near expected Mendelian frequencies and have no obvious physical or behavior abnormalities. Analysis of the distal femurs of six-month-old female mice revealed that knockout of CD68 resulted in increased trabecular bone that, nevertheless, has a reduced TMD. The mineral apposition rate of the knockout mice was increased, and this may relate to the observed decrease in trabecular TMD. Rapid bone formation could lead to insufficient mineralization, and there are examples of this in the literature[24]. We also found that CD68^−/−^ osteoclasts differentiated *in vitro* demonstrated aberrant morphology including accumulation of abnormal intracellular vesicles and increased sensitivity to detachment forces. In addition, osteoclasts that lack CD68 expression showed reduced bone resorption *in vitro*. These *in vitro* abnormalities along with histological TRAP staining of femur sections suggest that the increases in trabecular bone *in vivo* are due to decreased osteoclast activity, not number. A decrease in bone resorption with an increase in bone formation is unusual, as these processes are often paired. There are, however, instances where non-resorbing osteoclast can stimulate osteoblast activity[25], [26]. If this is the case for CD68 knockout mice, CD68 may prove to be a valuable target for an antiresorptive therapy that uncouples bone formation from bone resorption. The decreased trabecular TMD that results from the increase in MAR is a concern, and the biomechanical properties of bones from CD68 knockout animals should be assessed to determine any consequences of this reduction in TMD.

The *in vitro* phenotype of CD68^−/−^ osteoclasts is intriguing in that it recapitulates many of the abnormalities observed when the vesicular trafficking of osteoclasts is perturbed. Vesicular trafficking in osteoclasts is regulated by multiple factors including members of the Rab family of small GTPases [27]. Inhibition of individual Rab family members or their associated effectors results in varying degrees of defective vesicular trafficking and osteoclast dysfunction [28]–[31]. Lipid metabolism also contributes to normal vesicular trafficking in osteoclasts. Luegmayr et al. demonstrated that pharmacological depletion of cholesterol from cultured osteoclasts resulted in cells with large vacuole-like accumulations and an increased rate of apoptosis, and osteoclasts with deficient LDL uptake demonstrated similar defects that were rescued by cholesterol enrichment [32]. Beyond inhibition of cholesterol uptake, sequestration of cholesterol within osteoclast late endosomes is sufficient to disrupt vesicular trafficking and ruffled border formation by preventing cholesterol enrichment in the ruffled border [33]. While Rab function and lipid metabolism likely have discrete roles in osteoclast vesicular trafficking, there is certainly overlap between these two aspects. Rab functionality is often dependant upon prenylation, which is a form of lipid modification. Indeed, pharmacological inhibition of Rab geranylgeranylation results in vesicular accumulation and decreased osteoclast activity, and inhibition of farnesyl pyrophosphate synthase, an enzyme of lipid metabolism, is a proposed mechanism of osteoclast-inhibiting nitrogen-containing bisphosphonates which have been shown to disrupt vesicular trafficking [34]–[36].

The morphological defects we have observed in CD68^−/−^ osteoclasts, in light of the significance of lipid in vesicular trafficking and CD68’s known LDL binding properties, makes a role for CD68 in lipid processing and attractive possibility. While it has been demonstrated that CD68 is not a key contributor to LDL uptake, the uptake of lipid alone is not sufficient for the normal function of osteoclasts. Rather than LDL uptake, CD68 may have a role in delivery of lipids to their proper compartments or their addition to prenylated proteins. Determination of lipid distribution (i.e. cholesterol enrichment in the ruffled border) and GTPase prenylation in CD68^−/−^ osteoclasts will be an important step in investigating these possibilities. Another important vesicular trafficking event is the movement of bone-derived materials from the ruffled border to the apical surface via the process of transcytosis. As CD68 is arranged in a dome-like pattern proximal to the basolateral and apical membranes, it may play a role in the targeting of transcytosing vesicles to the apical membrane where their contents are released. Typing of the abnormal vesicles seen in CD68^−/−^osteoclasts to determine their origin (e.g. late endosomes, transcytotic vesicles) is another way of exploring CD68’s role in osteoclast trafficking. Coupled with studies of lipid distribution, these studies should further illuminate CD68’s function in osteoclasts and the mechanism of osteoclast dysfunction in its absence.

In our study, we have demonstrated the importance of CD68 expression in osteoclasts, and, in the process, produced a tool in the form of the CD68 knockout mouse for examining not only the contribution of CD68 to normal skeletal physiology, but also for answering questions pertaining to CD68’s function(s) in other tissues. Exploitation of CD68’s known properties have already been shown to have therapeutic value in preclinical studies of atherosclerosis, and future studies using CD68^−/−^ mice and their cells will hopefully shed light on new therapeutic opportunities.

## Materials and Methods

### Ethics Statement

Mice were maintained and the experiments involving mice were performed in accordance with the regulations of the University of Alabama at Birmingham Institutional Animal Care and Use Committee (animal protocol approval number: 100908911).

### Chemical and Reagents

Chemicals were purchased from Sigma (St. Louis, MO) unless indicated otherwise. DMEM (Cat No 10-013-CV), L-glutamine (25-005-CI) were purchased from Mediatech. Fetal bovine serum was purchased from Invitrogen (26140–079). Recombinant GST-RANKL was purified as previously described [37]. Mouse M-CSF was prepared from an M-CSF-producing cell line, CMG14–12, which was constructed and kindly provided by Dr. Sunao Takeshita [38]. Anti-CD68 antibody (rat monoclonal; FA-11), RPE-conjugated anti-CD68 antibody, and RPE-conjugated Rat non-immune IgG_2a_ were purchased from AbD Serotec (Raleigh, NC), anti-β-actin antibody (mouse monoclonal; AC-15) was purchased from Sigma, anti-Fc receptor antibody (clone 2.4 G2; sc-18867) was purchased from Santa Cruz Biotechnology (Santa Cruz, CA), β-tubulin (rabbit polyclonal; ab6046) was purchased from Abcam (Cambridge, MA), and Alexa-647 conjugated anti-rat-IgG (goat polyclonal; 4418) was purchased from Cell Signaling Technology (Boston, MA).

### Western immunoblotting

Cultured cells were lysed using cold cell lysis buffer with protease inhibitor cocktail (Cell Signaling Technology; 9803, 5871). Lysates were spun down to pellet insoluble precipitates and supernatant was transferred to clean tubes. Protein concentrations were measured by the Bradford method. 4X sample buffer (250 mM Tris; 140 mM sodium dodecyl sulfate [SDS]; 30 mM bromophenol blue; 2% β-mercaptoethanol; 40% glycerol) was added to protein solutions to a final concentration of 1X, and samples were heated at 95°C for 10 min to denature proteins. Samples were stored at −80°C prior to use.

Proteins were separated by sodium dodecyl sulfate polyacrylamide gel electrophoresis (SDS-PAGE; 8.5% acrylamide: bis-acrylamide 30∶1) in running buffer (25 mM Tris; 25 mM glycine; 3 mM SDS) and transferred to nitrocellulose membranes in transfer buffer (50 mM Tris; 40 mM glycine; 1 mM SDS; 20% methanol) using a semi-dry transfer apparatus for 30 min at 23 V. Membranes were blocked with 5% milk in Tris-buffered saline with .1% Tween-20 (TBS-T) for 1 hour at room temperature. Membranes were incubated with primary antibody (CD68, 5μg/mL; β-actin, 1μg/mL; α-Tubulin, 1μg/mL) at 4°C overnight. Membranes were washed 3x for 10 minutes with TBS-T, incubated with horse radish peroxidase-conjugated secondary antibody (.1μg/mL) for 1 hour at room temperature, and washed 3x for 10 minutes with TBS-T. Antibody-labeled proteins were detected with enhanced chemiluminescence and X-ray film. Densitometric analysis of films was performed using ImageJ software.

### Flow cytometric analysis

All incubations and centrifugations were carried out at 4°C. 1×10^6^ cells were fixed in solution with 4% paraformaldehyde/PBS for 20 minutes. Fixed cells were washed 3× with PBS for surface staining or PBS +.5% saponin for total staining, and Fc receptors were blocked with anti-Fc receptor antibody for 30 minutes. Cells were then stained with either RPE-conjugated anti-CD68 antibody or non-immune IgG_2a_ for 45 minutes. Non-permeablized cells were stained with an RPE-conjugated anti-Actin antibody as a negative control for surface staining. After 3 more washes with PBS (+/−.5% saponin), cells were analyzed using a Becton Dickinson SLR II analytical flow cytometer. Flow histograms were generated using WinMDI.

### *In vitro* osteoclastogenesis

BMMs were isolated from mouse long bones and were maintained in α-minimal essential medium (α-MEM) containing 10% heat-inactivated FBS with M-CSF (220 ng/mL) as previously described [39]. To generate osteoclasts from BMMs, 5×10^4^ adherent cells per well were plated in 24-well tissue culture plates (7.5×10^3^ cells for 96-well plates) and cultured in presence of 44 ng/mL M-CSF and 100 ng/mL of purified GST-RANKL for 3–5 days. The osteoclastogenesis cultures were stained for TRAP activity with Leukocyte Acid Phosphatase Kit (Sigma; 387-A).

### Immunofluorescent staining of osteoclasts

BMMs (5×10^4^ cells per well) were seeded directly into the wells of 24-well tissue culture plates or onto bone slices within 24-well culture plates and cultured under either macrophage-maintaining or osteoclastogenic conditions (with M-CSF only or M-CSF + RANKL). Cells were fixed with 4% paraformaldehyde solution in PBS for 20 minutes at room temperature. Cells were permeabilized and blocked with a 0.2% BSA/0.1% saponin/PBS solution (PBBS). Cells were incubated with anti-CD68 or non-immune control IgG_2a_ at a concentration of 10μg/mL in PBBS for 2 hours. Cells were then washed three times with PBBS and incubated with 1∶400 Hoescht, 1∶200 Alexa-488 conjugated phalloidin, and 5μg/mL Alexa-647 conjugated secondary antibody for 45 min. Cells were again washed three times with PBBS before mounting with 80% glycerol in PBS. Stained cells were imaged with a Zeiss LSM 710 confocal microscope.

### CD68 knockout mouse generation

CD68 targeting vector was constructed using standard molecular cloning methods on a pBluescript-SK backbone. The targeting vector was electroporated into C57B6/J mouse embryonic stem cells, and transfected cells were positively selected with G418 and negatively selected with ganciclovir. Surviving clones were screened via PCR and Southern blotting. Positive clones were injected into albino C57B6/J blastocysts to generate CD68^+/−^/CD68^+/+^ chimeras. Electroporation and embryonic stem cell injection was carried out at the UAB Transgenic Mouse Facility. Male chimeras were bred to albino C57B6/J females, and black-coated offspring were genotyped using PCR. CD68^+/−^ siblings were mated to produce CD68^+/+^, CD68^+/−^, and CD68^−/−^ mice.

### Micro-computed Tomography (μCT)

Ethanol-fixed femurs were embedded in polymethylmethacrylate were scanned and using a ScanCo Medical µCT40 in the UAB Small Animal Phenotyping Core. The following parameters were measured using this system: BV/TV, Tb. N, Tb. Sp., trabecular TMD, cortical TMD, and cortical thickness.

### Histological analysis

Prior to sacrifice, mice were injected subcutaneously with 100μL 20 mM calcein in PBS once and again 7 days later to label mineralization fronts for dynamic histomorphometric analysis. Formalin-fixed femurs were decalcified and embedded in paraffin blocks for sectioning. Slide-mounted sections were stained for Tartrate Resistant Acid Phosphatase activity and counterstained with hemotoxylin. Osteoclast number per mm bone surface was quantified using BioQuant software. 70%-ethanol fixed femurs were embedded in plastic and were sectioned and stained with Goldner’s trichrome. Static and dynamic histomorphometric analaysis was performed using BioQuant software. Tissue processing and staining was carried out by the UAB Center for Metabolic Bone Disease Histomorphometry Core.

### *In vitro* bone resorption assay

*In vitro* pit-forming assays were preformed similarly as previously described [40]. 5×10^4^ adherent BMMs were seeded onto bone slices within the wells of a 24-well culture plate under osteoclastogenic conditions. Osteoclasts were allowed to differentiate and mature over 4 days followed by an additional 3 days of resorption. Osteoclastogenic medium was refreshed every 2 days. At the conclusion of the resorption culture, cells were removed from the bone slices using 0.25 M ammonium hydroxide and mechanical agitation. Slices were washed with water and glued to microscope slides for imaging using a Zeiss LSM 710 confocal microscope. Resorption area as a percent of total area was quantified from resulting images using Adobe Photoshop.

### Soluble TRAP microtiter assay

Quantification of relative osteoclast number *in vitro* was carried out using the microtiter method of Minkin et al [41], [42]. Osteoclasts were lysed in 100μL soluble TRAP assay buffer (50 mM Sodium Acetate pH 5.0, .5 mg/mL 4-Nitrophenyl phosphate disodium salt [Sigma; S0942], .5% Triton X-100 [Sigma; 93443], and 10 mM L-Tartaric acid [Sigma; T109]) and incubated at 37°C for 5 min. Reaction was stopped and completed by addition of 50 µL .1 M NaOH. Absorbance was read at 405 nm with a reference wavelength of 625 nm.

### Statistical Analysis

All graphs were generated using Microsoft Excel. P-values were calculated using a two-tailed Student’s t-Test.
